# Leveraging Emergent Social Networks to Reduce Sedentary Behavior in Low-Income Parents With Preschool-Aged Children

**DOI:** 10.1177/21582440211031606

**Published:** 2021-07-23

**Authors:** Sabina B. Gesell, Shari L. Barkin, Edward H. Ip, Santiago J. Saldana, Evan C. Sommer, Thomas W. Valente, Kayla de la Haye

**Affiliations:** 1 Wake Forest School of Medicine, Winston-Salem, NC, USA; 2 Vanderbilt University Medical Center, Nashville, TN, USA; 3 University of Southern California, Los Angeles, CA USA

**Keywords:** sedentary behavior, obesity, clinical trials, behavior strategies, Hispanics

## Abstract

This study tested the hypothesis that parents participating in a pediatric obesity intervention who formed social network ties with a parent in the intervention arm would engage in more daily physical activity and less sedentary behavior (after controlling for participant covariates). Data were collected at baseline, 12 months, and 36 months from 610 low-income parent-child pairs participating in an obesity prevention intervention for 3- to 5-year-old children. A network survey was used to identify social network ties among parents and accelerometers were used to measure parental physical activity and sedentary time. Longitudinal regression analyses tested effects of social network ties on parents’ physical activity and sedentary behavior. Compared with parents without a social network tie, having a tie with an intervention group participant was associated with a clinically meaningful 11.04 min/day decrease in parental sedentary behavior that approached statistical significance (95% confidence interval [Cl] = [−22.71, 0.63], *p* = .06). Social network ties among parents in a pediatric obesity prevention intervention were not clearly associated with reduced sedentary behavior among those parents at the traditional level of *p* = .05. The large effect size (over 77min per week improvement) suggests there might be potential importance of promoting new social networks in community-based health promotion interventions to elicit and support behavior change, but further examination is needed.

## Introduction

Obesity (Christakis & Fowler, 2007; Shoham et al., 2012; Valente et al., 2009; S. Zhang et al., 2018), physical activity (de la Haye et al., 2011; Gesell et al., 2012; J. Zhang et al., 2015), diet (de la Haye et al., 2013), and sleep (Mednick et al., 2010) spread predictably through existing family and friendship networks. Individuals befriend others with similar behavioral patterns, which tends to reinforce the behavior or weight norm (de la Haye et al., 2011; Valente et al., 2009). Thus, social networks may be crucial mediators in the onset, development, and maintenance of obesity, and weight-influencing behaviors (de la Haye et al., 2011; Gesell et al., 2012; Hammond, 2009; Shin et al., 2014; J. Zhang et al., 2015) and interventions that operate on and through social networks may be effective in changing weight-influencing behaviors. Few studies have examined how to engineer in-person social networks to increase physical activity (Rovniak et al., 2013), diet, and weight loss (Hunter et al., 2019). This study examines one social network approach: expanding an individual’s social network with a person engaged in a healthy lifestyle intervention.

The terms “social networks” and “social support” are often used interchangeably, but are separate constructs (Gesell, Bess, Barkin, 2012). Social support is defined as the support (emotional, informational, or tangible) that a person perceives from others which can increase self-efficacy and the adoption of health behaviors. Social support can also serve as a buffer to stressors or illness. Social networks, in contrast, measure the presence or absence of friendships and other affective or instrumental relationships (which may or may not provide support) (Smith & Christakis, 2008). Both influence health behaviors. This article focuses on social networks that form when people are brought together for a behavior change intervention in person (not online).

In previous pilot work, this team showed new social network ties developed among parent participants within the first months of a pediatric obesity intervention, and these extended beyond the intervention context (Gesell et al., 2016). Moreover, these new social network ties were associated with increased perceptions of group cohesion among intervention participants, measured by a sense of belonging and feelings of morale (Bollen & Hoyle, 1990). Also, parents formed social network ties based on child body mass index (BMI), such that mothers selectively formed new friendships with mothers whose children had similar body types (Gesell, Bess, Barkin, 2012). However, that study lacked statistical power to examine whether these ties affected parental behaviors.

The purpose of this article was to test, within a larger social network and behavioral intervention, whether social network ties among study participants were associated with daily sedentary behavior and physical activity change. People who are well integrated into a community generally adopt behaviors earlier than those who are less integrated (Coleman et al., 1966; Rogers, 1995), suggesting participants who establish social connections to intervention group members may be more likely to adopt the promoted health behaviors. This study tested the hypothesis that parents who formed social connections with participants *in the intervention arm* would engage in less sedentary behavior and more physical activity, compared with parents who did not form such ties.

## Method

### The GROW Intervention

Growing Right Onto Wellness (GROW) was one of the four National Institutes of Health’s (NIH) Childhood Obesity Prevention and Treatment Research (COPTR) Consortium obesity prevention randomized controlled trials in children. Its methods (Po’e et al., 2013) and effects on child outcomes (Barkin et al., 2018) are published. That study is registered at ClinicalTrials.gov (NCT01316653). Briefly, GROW was a 3-year randomized controlled trial to test the effect of a family-based, community-based pediatric obesity prevention intervention. The primary outcome was childhood growth trajectories. GROW randomized parent–preschool child pairs to either (a) a behavioral intervention that used group educational sessions and motivational interviewing to build skills for healthy lifestyles for both children and adults (GROW Healthier) or (b) a school readiness comparator group (GROW Smarter). The comparator condition was administered to both control and intervention participants at separate times, so the only difference between the two groups was the delivery of the GROW Healthier content. The intervention group promoted healthy weight and related behaviors including increasing daily physical activity and reducing sedentary behavior in parents and their preschool-aged children. There were three intervention phases: (a) the intensive phase delivered in a group format where small, consistent groups of participants met 12 times, once a week, for 90 min each time, over the first 3 months; (b) the maintenance phase, which included monthly phone-call coaching over 9 months (12-month timepoint); and (c) the sustainability phase, which included cues to action to use the surrounding built environment for health over 24 months (36-month timepoint). Parents in the intervention group built skills in nutrition, physical activity, and parenting, concurrently forming new social networks, and were given memberships and introductions to their local recreation center. Participants were taught goal-setting, self-monitoring, and problem-solving skills to facilitate sustainable behavior change. The curriculum used low-health literacy communication techniques to deliver key health messages (White et al., 2013). Integrated within the GROW Healthier intervention was the intentional building of new social networks among intervention group participants during the intensive phase (Gesell et al., 2013). Valente (2012) describes several types of network interventions that intentionally leverage social networks to encourage health behavior change. GROW was an induction intervention: It intentionally created peer-to-peer interaction to spread new ideas and behaviors within the intervention group. There was no network building component for the GROW Smarter comparator group.

### Sample

The GROW trial focused on parents as agents of change (Faith et al., 2012). GROW used a rolling recruitment strategy to enroll 610 (304 intervention, 306 control) parent–child pairs in low-income communities. Eligibility criteria included the following: parent aged ≥18 years; parent had at least one child 3 to 5 years old with BMI ≥50th and <95th percentile; parent spoke English or Spanish; parent had consistent phone access; parent lived or worked within 5 miles of the participating community centers in Nashville, TN; parent and child were healthy and lacked medical conditions necessitating limited physical activity as evaluated by a pre-screen; and parent and child were considered underserved, as indicated by self-report that either the index parent or someone in the household was eligible to participate in at least one government-subsidized program for healthcare insurance or food assistance.

### Data Collection

Social network, physical activity, and sedentary behavior data were collected at three timepoints (baseline, 12 months, and 36 months), from June 2014 to July 2017. The Vanderbilt University Medical Center Institutional Review Board (IRB No. 120643) and an NHLBI-appointed Data and Safety Monitoring Board approved the study protocol and routinely evaluated both participant safety and protocol adherence. Using participants’ language of choice, written informed consent was obtained by bilingual data collectors using an enhanced low-literacy approach (Heerman et al., 2015). Trained data collectors were blinded to intervention assignment. Data collection occurred at local community centers or at participants’ homes (as participants preferred). The REDCap database system was used for data collection and management (Harris et al., 2009).

### Measures

#### Social network.

To identify directed social network ties among adult study participants during the study, all participants responded to the following name generator question at each assessment: “Please provide the names of up to 7 people you know and talk to from GROW (this can include anyone in GROW Smarter or GROW Healthier).” No roster was provided to participants, and there were no other restrictions on who they could nominate. Ties could have formed between any pair of intervention participants and any pair of control participants because there were structured events for all participants in each study group. Intervention and control group members were always kept separate to avoid contamination. However, participants lived in the same city and connections could have existed or formed over time between individuals in the two study groups nonetheless. Participants who did not nominate anyone were included as not having a tie (see “Social Network Analysis” section below). Ties among pairs of study participants were directed, at a given observation, and these lists of social network ties were used to compute network statistics. Data were collected in an individual format with privacy protections provided. Because this was a low-literacy population, data collectors read the survey questions aloud (in participants’ preferred language) and entered respondents’ answers directly into REDCap.

#### Physical activity.

ActiGraph GT3X+ accelerometers were used to measure sedentary behavior and physical activity. Parents were instructed to wear the lightweight monitor on the right hip continuously for seven complete days. The minimum valid wear-time criteria were 4 days (3 weekdays and 1 weekend day) with at least 6hr of wear time between 5:00 a.m. and 11:59 p.m. If these criteria were not met, participants were asked to repeat the ActiGraph measurements. Matthew’s validated cut points were used to define three outcome variables: sedentary behavior (≤100), light physical activity (>100 to ≤2,100), and moderate-to-vigorous physical activity (MVPA) (>2,100 counts/1 min) for adults (Matthew, 2005; Matthews et al., 2008). As part of the NIH’s Childhood Obesity Prevention and Treatment Research Consortium, experts created wear-time criteria. Their consensus was as follows: Accelerometry data should be collected using the GT3X + monitor worn on the right hip for seven complete days, including sleeping and during water activity (e.g., bathing, swimming, showering). The ActiGraph GT3X + assesses acceleration in three individual orthogonal planes using a vertical axis, horizontal axis, and a perpendicular axis. The GT3X + was set to a 40-Hz frequency. Importantly, as part of the accelerometry adherence and wear-time validation protocol, and before physical activity variables (including wear time and valid wear days) were derived, an algorithm by Choi et al. (2011,^2012^) was applied to remove nonwear time based on specific patterns of consecutive zero counts. In addition, participants in this study had very high wear-time compliance with a mean daily total wear time of approximately 17 hr (see Table I) out of the 19 hr possible.

#### Covariates potentially related to physical activity.

Covariates were assessed using validated measures and included age, sex, pregnancy status (weeks pregnant and weeks since birth), food insecurity (Gulliford et al., 2004), depression (Radloff, 1977), study randomization, intervention dose, timepoint, mean daily accelerometer wear time, and BMI.

### Analytic Approach

#### Social network analysis.

Social network analysis integrates the relational data (ties between parents) and participant-level data (i.e., their study group) to compute summary statistics of participants’ social network ties, based on their nominations. Three mutually exclusive categorical variables were computed for the primary independent variable *Tie Type* at each timepoint: at least one tie to someone in the intervention group, regardless of ties to the control group [Intervention tie]; at least one tie to someone in the control group but no ties to the intervention group [Control tie]; no tie (reference category) [No tie]. These categories were used to test whether having at least one tie to an intervention participant was benficial, compared with having zero ties or ties only to study participants the control group.

#### Longitudinal regression modeling.

Longitudinal regression analyses using generalized estimating equations were used to test relationships of social network ties categories to parent sedentary behavior and physical activity. The longitudinal model assumes a first-order autoregressive (AR1) covariance structure for observations over time. In other words, two consecutive observations were modeled as correlated and the strength of correlation was assumed to be constant over time. Sensitivity analyses were conducted to examine the viability of the AR1 assumption. Goodness-of-fit metrics included the Quasilikelihood under the Independence Model Criterion (QIC), its extension QICU (Pan, 2001) (relative fit metrics), and marginal *R*^2^ (Zheng, 2000) (absolute fit metric). There were three outcome variables: minutes of MVPA, minutes of light physical activity, and minutes of sedentary behavior. The primary independent variable was the three categories of participants’ social network ties.

First, descriptive statistics were run to characterize the data set. Next, three longitudinal linear regression analyses (one for each outcome variable) were conducted. Observations across timepoints were treated as repeated measurements. In addition, each longitudinal analysis assumed an autoregressive dependence of the outcome between timepoints. Covariates included intervention status, parent age and gender, timepoint, weeks since birth, weeks pregnant, intervention dose, food insecurity, depression, mean daily accelerometer wear time, and parental BMI.

Because pregnancy could change the trajectories of the adult outcome variables, at each timepoint of measurement, two variables—weeks since birth and weeks being pregnant—were included. To adjust physical activity estimates for variation in wear time and to provide results in the easily interpreted and clinically useful metric of mean minutes per day, the amount of time participants wore the accelerometer (wear time) was adjusted for in the analyses, as is standard practice (Evenson et al., 2019).

#### Missing values.

In general, missing values were treated as missing-at-random (MAR), meaning missing values could depend on predictors but whether a data point is missing could not depend on the value of the missing data point. When the outcome measurement at a given timepoint was missing, the specific data point was not included in the longitudinal model. Gender was never missing and not imputed. There was systematic missingness in the parental physical activity data because pregnant women stopped wearing their accelerometer over the course of their pregnancy. In addition, there was a delay in the administration of the social network survey at the start of the trial and network tie data were not collected from approximately 23% of the participants at baseline. As a result, only missing baseline network data were multiply imputed (5 times) using an imputation model in which ties were modeled as a function of study randomization, parent age, and number of MVPA and resting/sedentary minutes per day. Results from analyses of the imputed network tie data sets fully accounted for the uncertainty due to imputing missing values.

All analyses were performed using SAS 9.4, including the repeated measurement analysis program PROC GENMOD. All tests were two-sided with *p*=.05. Reciprocity, used as a descriptive statistic, was calculated using the iGraph v0.7.0 package in R. The imputation model was implemented via SAS 9.4 PROC MI and analysis of the multiply imputed data set was conducted using PROC MIANALYZE.

## Results

### Sample Characteristics

Participant characteristics are shown in Table 1. At baseline, parents were 98.4% female (600/610), 91.1% Hispanic (556/610), and had a mean BMI of 29.8 (*SD*=6.2) kg/m^2^. This was a low-income cohort with low levels of formal education: Of the portion who responded, 78% (346/442) had a household income of less than US$25,000, and 61% had less than a high school degree or equivalent (374/610). Among the 561 parents who met the minimum accelerometer wear-time criteria at baseline, the mean daily wear time was 1,009.2 (*SD*=155.4) min, sedentary behavior was 476.2 (*SD*=126.4) min, light physical activity was 483.8 (*SD*=102.1) min, and MVPA was 49.2 (*SD*=39.6) min. Among these 561 parents, 11 (2%) were pregnant at baseline, 11 (2.5%) were pregnant at 12 months, and 0 (0%) were pregnant at 36 months.

### Social Network Ties

At all three timepoints, the social network was sparse: Most participants had no ties (Table 2). The average number of ties per participant is shown in Table 3.

However, after baseline, participants in the intervention arm tended to form ties with others in that arm, a trend that persisted over time (increased from 16.4% at baseline to 28.1% and 37.6% at 12 and 36 months, respectively), whereas participants in the control group formed ties at a notably slower rate and had comparatively fewer ties to participants in the intervention (increased from 10.1% at baseline to 12.0% and 17.1% at 12 and 36 months, respectively).

### Longitudinal Regression Modeling

Marginal *R*^2^ values for different outcomes MVPA, sedentary behavior, and light physical activity were, respectively, .08, .53, and .48. QIC and QICU were close for different covariance structure (e.g., QIC ranged from 1,449 to 1,451 for MVPA for four commonly used covariance structures including AR1 which was used for reporting; Table 4).

Compared with having no tie, having at least one tie with an intervention group member predicted an 11.04 min/day decrease in parent sedentary behavior (95% confidence interval [Cl] = [−22.71, 0.63], *p* = .06; Table 5). This effect was not statistically significant, but the effect size was notable. The effect of having an intervention tie on parent MVPA and light physical activity, respectively, was not statistically significant (MVPA increase of 3.26 min/day, 95% Cl = [−1.77, 8.28], *P*=.20, and light physical activity increase of 7.86 min/day, 95% Cl = [−2.10, 17.82], *p*=. 12; Tables 6 and 7). While the 95% CIs include the potential for a null or very small negative relationship, they do not rule out a potentially beneficial and clinically meaningful change.

## Discussion

### Main Findings

While not statistically significant at the .05 level, the potential magnitude of the effect is notable and should be considered clinically meaningful, especially compared with other behavioral studies which have demonstrated statistically significant, but much smaller effects (Sullivan & Feinn, 2012). Our family-based pediatric obesity prevention intervention facilitated the building of social network ties, particularly among intervention arm participants, and those ties may be associated with reductions in parental sedentary behavior. Parents (regardless of group assignment) who had a social tie to at least one intervention participant spent a *marginally* significant adjusted average of 11.04 min less time in sedentary behavior per day, compared with parents without a tie (*p*=.06). While the 95% CIs include the potential for a null or very small negative relationship, they do not rule out a potentially beneficial and clinically meaningful change.

Although not statistically significant, additional results suggest parents may have shifted most of that sedentary time to light-intensity physical activity (7.86 min/day increase), rather than MVPA (3.26 min/day increase).

The reduction in sedentary behavior of 11.04 min/day (77.28 min/week) is clinically meaningful because replacing sitting time with light-intensity physical activity reduces the risk of all-cause mortality. The 2018 Physical Activity Guidelines Advisory Committee (2018) reported a strong relationship between sedentary time and the risk of all-cause mortality in adults. Clearly, reducing sedentary behavior is healthy. However, to date there are no evidence-based guidelines for total sedentary time or the frequency with which sedentary time should be interrupted with physical activity each day.

A major tenet of the social-ecological model—namely, the layering of strategies at multiple levels of individuals’ social and ecological environments may be synergistic—could explain the potential reduction in sedentary behavior. The GROW trial strove to change parent behavior by intervening on the parent in the context of a new social network and connecting them to a built environment that supports physical activity. The social network ties that parents shared with study participants who received the intervention, most of which did not precede the study, appeared to have influenced their own behavior positively.

Parents in both groups benefited from ties with parents in the intervention arm. Parents in both groups who knew and talked to at least one person in the intervention group adopted and sustained a reduction in sedentary behavior during the 3-year study period compared with parents without such ties. The following might be underlying mechanisms. Social connection to someone making healthy behavior changes provides a new source of health information, norms, or opportunities to engage in healthy behaviors (Valente, 2010). This social influence effect could explain our findings, especially in the control arm. Among parents receiving the intervention, those who were engaged in making healthy changes together to reduce health risks for themselves and their children may have generated communal coping processes that foster interpersonal communication, support, and co-engagement in solutions and behaviors to reduce their collective risk (Afifi et al., 2006). When these processes are elicited in social groups receiving a health behavior intervention, successful behavior change is more likely than if attempted at an individual level (de Heer et al., 2017).

Because our results were not statistically significant, we must exercise caution in overinterpreting the findings. No effect of social network tie on adult physical activity would align with the most recently conducted comprehensive review of social network interventions on health behaviors. That paper highlighted that the need to further investigate the effectiveness of social network interventions on health behaviors found no significant intervention effects for several outcomes of single studies, including physical activity, diet, and weight loss (Hunter et al., 2019). That paper also concluded that the best way to apply social network intervention approaches to various health interventions remains unknown. A reviewer of this paper suggested examining the effect of more than one connection—which theoretically would strengthen the reinforcement of the desired behavior change. However, given the scarcity of the network this analysis was not conducted.

Interventionists documented stories from adult participants throughout the GROW study. Qualitative analysis of these stories demonstrated the importance of social connections, within and between families, as a key driver of health behavior changes from their perspective. Among stories addressing community center use for physical activity, barriers included time pressures and transportation. Strategies included social connections that provided social support (specifically, accountability, companionship, and generating solutions to problems) and enabled families to be physically active together (such as attending the Family Swim nights at the local community center due to an invitation from a fellow GROW family who lives in the neighborhood).

### Strengths

The primary innovation in this article is the embedding of social network analysis within a randomized controlled trial. The translation of network science into effective interventions for obesity has been slow, mainly because longitudinal data sets with social network data and objective measures of weight-influencing behaviors are rare and expensive to create. The GROW trial allowed us to leverage a richly characterized cohort and create such a data set. It was not financially feasible to capture a complete picture of participants’ social networks, and certainly existing friendships and family members likely contribute to daily physical activity. Nonetheless, this study highlights how obesity interventions that intentionally leverage social networks as a mechanism for spreading knowledge, norms, and behaviors may be effective in improving health across communities. The focus on Latina women is a strength of this study given that this population has been traditionally marginalized and excluded in research.

### Limitations

The trial had rolling recruitment over 1.75 years. This added complexity to the social network data collection: It was not possible to use a roster to collect social network data, which might have prevented the complete capture of all existing social network ties, particularly at baseline. In addition, the survey administration was delayed at the start of the trial, reducing the amount of network data captured in the first months of the trial. Conversely, it is highly unlikely that existing social network ties were overestimated.

Accelerometry completion rates were high (75%, *n*=456/610 at 36 months), but limited accelerometry data were gathered for pregnant participants and contributed to missing data in a predictable manner. Using accelerometers that can be worn somewhere other than the waist may improve completion rates. There was little differential dropout (78% and 72% for intervention and control groups, respectively), supporting high internal validity. Results are likely generalizable to other economically disadvantaged, predominantly Hispanic mothers with preschool-aged children.

Although the original trial was sufficiently powered to detect meaningful effect sizes on the primary outcome (child BMI trajectory), these secondary analyses were based on a smaller sample size (i.e., those with sufficient social network and accelerometry data), which likely reduced power.

The study sample was very homogeneous in that it was predominantly Latina women, who were predominantly low income. Race was not included in the models. Food security was included in the models because it can be robust proxy for income and/or education. However, given the homogeneity of the sample, the study was not well suited to test how these demographic variables affect (e.g., moderate) the findings.

### Future Research

This study adds to this literature by showing the potential beneficial effects of social network ties on the amount of time parents spend in sedentary behavior within the context of a pediatric obesity prevention intervention that brought together a new social network. Network interventions (Valente, 2012)—which intentionally leverage social networks to change specific behaviors and improve health—have successfully prevented substance use (Valente et al., 2007) and reduced HIV risk behavior (Kelly et al., 1991). There is growing interest in using this approach to increase physical activity (Hunter et al., 2015). This study provides empirical evidence that induction interventions are a promising avenue for eliciting and sustaining reduced sedentary behavior among low-income parents. Finally, this study shows the feasibility of capturing network data within a clinical trial and the potential of creating more data sets that have both social network and behavior data by adding network measures to interventions that have peer-to-peer interaction. Future research studies would benefit from being adequately powered to answer important questions about covariates that may play a role in moderating the relationships between networks and behavior (e.g., ethnicity/race, and socioeconomic status [SES]).

## Conclusion

Within a pediatric obesity prevention intervention, parents who had social network ties to parents who received the intervention reduced their sedentary behavior. Having a social tie to an intervention group participant resulted in a clinically meaningful 11.04 min/day decrease in parent sedentary behavior (about 77.28 min/week) during a 3-year observation period, compared with parents who did not form a tie. Future studies can embed social network analysis within a randomized trial and can potentially leverage social network ties to elicit and sustain reduction in adult sedentary behavior.

## Figures and Tables

**Table I.** Parental Characteristics in the GROW Trial at Baseline.

Variable	Intervention (*n* = 304)	Control (*n* = 306)	Total (*N* = 610)

Characteristics			
Gender			
Male	4 (1.3%)	6 (2.0%)	10 (1.6%)
Female	300 (98.7%)	300 (98.0%)	600 (98.4%)
Age (years)	32.5 (6.2)	31.6 (5.8)	32.1 (6.0)
Body mass index (kg/m^2^)	29.8 (6.2)	29.4 (5.3)	29.6 (5.8)
Race/ethnicity			
Non-Hispanic White	4 (1.3%)	5 (1.6%)	9 (1.5%)
Non-Hispanic Black	19 (6.3%)	20 (6.5%)	39 (6.4%)
Hispanic Mexican	183 (60.2%)	204 (66.7%)	387 (63.4%)
Hispanic non-Mexican	95 (31.3%)	74 (24.2%)	169 (27.7%)
Non-Hispanic multiracial	3 (1.0%)	3 (1.0%)	6 (1.0%)
Center for Epidemiological Studies-Depression score^a^ (*N* = 609)	10.4 (9.2)	9.3 (8.9)	9.9 (9.0)
Food insecurity level^b^ (*N* = 606)			
Food secure (0–1)	165 (54.6%)	183 (60.2%)	348 (57.4%)
Food insecure without hunger (2–4)	86 (28.5%)	87 (28.6%)	173 (28.5%)
Food insecure with hunger (5–6)	51 (16.9%)	34 (11.2%)	85 (14.0%)
Household income			
US$14,999 or less	85 (28.0%)	89 (29.1%)	174 (28.5%)
US$ 15,000-US$24,999	90 (29.6%)	82 (26.8%)	172 (28.2%)
US$25,000-US$34,999	39 (12.8%)	37 (12.1%)	76 (12.5%)
US$35,000-US$49,999	7 (2.3%)	9 (2.9%)	16 (2.6%)
US$50,000- US$74,999	2 (0.7%)	2 (0.7%)	4 (0.7%)
Don’t know or no answer	81 (26.6%)	87 (28.4%)	168 (27.5%)
Education			
High school incomplete	182 (59.9%)	192 (62.7%)	374 (61.3%)
High-school degree or equivalent	122 (40.1%)	114 (37.3%)	236 (38.7%)
Accelerometry (min), *N*=561			
Mean daily total wear time	1,004.2 (156.7)	1,014.1 (154.1)	1,009.2 (155.4)
Mean daily moderate/vigorous physical activity	47.4 (38.2)	50.9 (41.0)	49.2 (39.6)
Mean daily light physical activity	479.8 (103.4)	487.7 (100.9)	483.8 (102.1)
Mean daily sedentary behavior	477.0 (130.0)	475.5 (123.0)	476.2 (126.4)

*Note*. Data are expressed as mean (*SD*) or frequency (%). Body mass index (calculated in weight in kilograms divided by height in meters squared).

All randomized participants are included. The correct sample size is indicated for variables with missing data. Physical activity measured with triaxial accelerometers. Individual wear time was averaged across valid wear days to produce a mean daily wear time and time in each physical activity category. GROW = Growing Right Onto Wellness.

a The CES-Depression survey consists of 20 questions. Scores range from 0 to 60, and higher scores indicate greater depressive symptoms (Radloff, 1977).

b Standard 6-question short-form survey for classifying households into food security status levels. The scale ranges from 0 to 6, and higher scores indicate greater food insecurity. Survey instructions were used to code raw scores into categories (Gulliford et al., 2004).

**Table 2.** Participants’ Social Network Tie Categories at Each Timepoint.

	Intervention	Control	Total
Variable	*n* (%)	*n* (%)	*N* (%)

^a^Baseline	*N* = 304	*N* = 306	*N* = 610
Tie type^b^			
Intervention tie	50 (16.4)	31 (10.1)	81 (13.3)
Control tie	17 (5.6)	14 (4.6)	31 (5.1)
No tie	237 (78.0)	261 (85.3)	498 (81.6)
12 months	*N* = 263	*N* = 259	*N* = 522
Tie type^b^			
Intervention tie	74 (28.1)	31 (12.0)	105 (20.1)
Control tie	16 (6.1)	22 (8.5)	38 (7.3)
No tie	173 (65.8)	206 (79.5)	379 (72.6)
36 months	*N* = 258	*N* = 245	*N* = 503
Tie type^b^			
Intervention tie	97 (37.6)	42 (17.1)	139 (27.6)
Control tie	26 (10.1)	33 (13.5)	59 (11.7)
No tie	135 (52.3)	170 (69.4)	305 (60.6)

a Values are representative of a single imputation for baseline data. Data at subsequent timepoints were not imputed.

b Definitions: Intervention tie: at least one tie to an intervention participant Control tie: at least one tie to a control participant, but no ties to an intervention participant. No tie: no tie to a participant.

**Table 3.** Average Number of Social Network Ties Participants Reported Having With Other Study Participants, at Baseline, 3-, 12-, and 36-Month Timepoints.

	Intervention				Control			
Variable	0	3	12	36	0	3	12	36

Ties	0.2	0.8	0.4	0.5	0.1	0.2	0.2	0.3
Intervention ties	0.1	0.7	0.3	0.3	0	0	0	0
Control ties	0.1	0.1	0.1	0.2	0.1	0.2	0.2	0.3

**Table 4.** Longituinal Model Fit Metrics.

Variable	Covariance structure	Marginal *R*^2a^	QIC^b^	QICu^b^

MVPA	AR	.08	1,449	1,445
	Exchangeable	.08	1,449	1,445
	Independent	.09	1,451	1,445
	Unstructured	.08	1,449	1,445
Sedentary behavior	AR	.53	1,455	1,445
	Exchangeable	.53	1,454	1,445
	Independent	.53	1,458	1,445
	Unstructured	.53	1,453	1,445
Light activity	AR	.48	1,457	1,445
	Exchangeable	.47	1,445	1,445
	Independent	.48	1,459	1,445
	Unstructured	.47	1,456	1,445

*Note.* QIC = Quasilikelihood under the Independence Model Criterion; MVPA = moderate-to-vigorous physical activity.

a Derived from using SAS macro %SelectGEE in Teng et al. (2009).

b Reported directly from result in SAS v9.4 PROC GENMOD.

**Table 5.** Longitudinal Analysis of Parental Daily Sedentary Behavior (in Minutes).

Variable	Category	Estimate	Lower 95% Cl	Upper 95% Cl	*p* value

Network tie type^a^	Intervention tie	−11.04	−22.71	0.63	.06
	Control tie	−10.59	−28.56	7.37	.25
Study randomization^a^	Intervention	−6.72	−46.55	33.12	.74
Baseline age (years)		−2.29	−3.57	−1.01	<.01
Gender^a,b^	Female	−49.70	−96.66	−2.75	.04
Timepoint (months)		−0.08	−0.36	0.20	.57
Weeks since birth for pregnant women		−0.22	−0.47	0.03	.08
Weeks pregnant		2.38	0.18	4.57	.03
Number of intervention sessions attended		0.86	−2.65	4.36	.63
Food security		1.88	−6.73	10.50	.67
Center for Epidemiologic Studies–Depression score		0.40	−0.20	0.99	.19
Mean daily accelerometer wear time (min)		0.54	0.51	0.57	<.01
BMI (kg/m^2^)		2.97	1.63	4.31	<.01

*Note*. Cl = confidence interval; BMI = body mass index.

a Reference groups are No tie, Control, and Female.

b Only 1.6% of participants were male.

**Table 6.** Longitudinal Analysis of Parental Daily Moderate-to-Vigorous Physical Activity (in Minutes).

Variable	Category	Estimate	Lower 95% Cl	Upper 95% Cl	*p* value

Network tie type^a^	Intervention tie	3.26	−1.77	8.28	.20
	Control tie	3.57	−4.51	11.65	.39
Study randomization^a^	Intervention	3.63	−15.59	22.85	.71
Baseline age (years)		0.19	−0.33	0.71	.48
Gender^a^	Female	−1.89	−15.78	11.99	.79
Timepoint (months)		0.14	0.02	0.26	.02
Weeks since birth for pregnant women		−0.04	−0.13	0.04	.33
Weeks pregnant		−0.96	−1.68	−0.24	<.01
Number of intervention sessions attended		−0.48	−2.14	1.18	.57
Food security		−2.12	−5.94	1.70	.28
Center for Epidemiologic Studies–Depression score		0.09	−0.19	0.36	.54
Mean daily accelerometer wear time (min)		0.03	0.02	0.04	<.01
BMI (kg/m^2^)		−0.25	−0.69	0.18	.26

*Note.* CI = confidence interval; BMI = body mass index.

a Reference groups are No tie, Control, and Female.

**Table 7.** Longitudinal Analysis of Parental Daily Light Physical Activity (in Minutes).

Variable	Category	Estimate	Lower 95% Cl	Upper 95% Cl	*p* value

Network tie type^a^	Intervention tie	7.86	−2.10	17.82	.12
	Control tie	7.65	−7.81	23.11	.33
Study randomization^a^	Intervention	2.92	−28.04	33.87	.85
Baseline age (years)		2.11	1.06	3.15	<.01
Gender^a^	Female	51.58	−0.76	103.92	.05
Timepoint (months)		−0.06	−0.30	0.18	.62
Weeks since birth for pregnant women		0.27	0.04	0.49	.02
Weeks pregnant		−1.43	−3.38	0.51	.15
Number of intervention sessions attended		−0.37	−3.12	2.38	.79
Food security		0.28	−6.96	7.51	.94
Center for Epidemiologic Studies–Depression score		−0.45	−0.94	0.04	.07
Mean daily accelerometer wear time (min)		0.43	0.40	0.45	<.01
BMI (kg/m^2^)		−2.75	−3.88	−1.63	<.01

*Note*. CI = confidence interval; BMI = body mass index.

a Reference groups are: No tie, Control, and Female.
